# Analgesic Treatment and the Patients’ Opinion on the Hospital Emergency Department

**DOI:** 10.3390/healthcare10040623

**Published:** 2022-03-25

**Authors:** Michał Wójcik, Anna Rogalska

**Affiliations:** 1 Emergency Department, Regional Hospital, 43-316 Bielsko-Biala, Poland; wwojcikmichal@gmail.com; 2 Department of Economics and Management in Health Care, School of Health Sciences in Bytom, Medical University of Silesia, 40-055 Katowice, Poland

**Keywords:** pain assessment scales, emergency department (ED), pain management, analgesia, the effect of therapy, patient satisfaction, healthcare

## Abstract

Aim: The aim of the study was to analyze whether a patient’s opinion is related to the effect of analgesic treatment. Methods: The study was conducted using a survey questionnaire among adult patients admitted to the hospital emergency department in March 2021. The Numerical Rating Scale (NRS) was used to assess pain. Patients were asked to rate the intensity of pain during their stay in the emergency department in three situations: (1) at admission; (2) during the stay; and (3) upon discharge. The relationships between qualitative variables were assessed by the chi-squared test. Significance was set at *p* < 0.05. Results: There was no statistical dependence between the patient’s opinion about the medical institution and results of the effectiveness of analgesic treatments (*p* = 0.056). The highest percentage of patients satisfied with the received treatment were those who did not feel pain during ED discharge (94.12%), and the lowest were those who complained of severe pain during ED discharge (63.91%). The average mark for the functioning and organization of the emergency department was 7.44 (±2.04). Only 54 patients (29.83%) had taken pain medication before deciding to visit ED. Conclusions: No statistical dependency between the effect of the analgesic treatment and the patient’s opinion has been observed. The majority of patients with pain discomfort visit emergency departments without looking for consultation in other locations or without taking analgesics. In the considered institution, patients were satisfied with the analgesic treatment, staff performance, and with the organization of the department.

## 1. Introduction

Although healthcare needs have developed from acute to chronic conditions in recent decades, the number of patients admitted to emergency departments (EDs) has increased worldwide, both in public and private healthcare systems [1,2]. Emergency departments are characterized by the fact that they are the fastest-operating, most intense, and most complicated department in a hospital. The main goals of EDs are to save lives, assess patients’ needs for urgent interventions, and provide treatment and care [3]. Almost 80% of the reasons for referral to an emergency department (ED) are pain [4,5]. As a result, emergency medicine physicians often need to use multimodal methods to achieve safe and effective pain control [6]. Despite the growing number of patients, it is important to maintain the highest quality of care [7]. Patient satisfaction may be an indicator of the quality of services provided in the emergency department [8]. Pain management in the emergency department is one of the qualitative indicators of care. The effectiveness of this treatment may influence the assessment of care in EDs [9]. The measurement of patient satisfaction is a helpful and necessary procedure [10].

## 2. The Study

### 2.1. Aims

The aim of the study was to answer the question of whether patients’ opinions about the emergency department were related to the effects of pain treatments. The second goal was to investigate the intensity of pain and whether the patients had used pain medication before arriving at the ED.

### 2.2. Design

A study using a questionnaire was conducted among patients who used the medical services of one multidisciplinary hospital emergency department in March 2021 in Poland. The Numerical Rating Scale (NRS) was used to assess pain. Patients rated their pain from 0 to 10: zero represented ‘no pain at all’, whereas the upper limit represented ‘the worst pain ever possible’ [11]. According to the intensity of the pain, it was divided into 3 grades:

- Mild (NRS 1–3);
- Moderate (NRS 4–6);
- Strong (NRS 7–10) [12].

Patients were asked to rate the intensity of pain during their stay in the emergency department in 3 situations: (1) at admission; (2) during the stay; and (3) upon discharge.

### 2.3. Population

The study was conducted in a contact manner, maintaining voluntary anonymity, as well as with respect for the patient’s rights, including maintaining medical secrecy. Patients gave oral informed consent for their involvement in the study. The inclusion criteria for the study included the following factors: (a) using health services in the ED in March 2021; (b) the patient’s state of health that allowed them to take part in the study; (c) full logical contact of the patient and orientation as to the place, time and person; (d) ensuring that the patient was not under the influence of narcotics or designer drugs; and (e) patients aged >18 years. The following data demographics were entered into the database: age, gender, place of residence, and level of education. A total of 218 patients participated in the study, but due to the lack of proper completion of the questionnaire, 12 questionnaires were excluded from the study.

### 2.4. Data Analysis

A group of 206 people was involved in the creation of the database. Statistical analysis was performed using the STATISTICA program, version 13.1 (StatSoft PL, Cracow, Poland). For the analysis of sociodemographic characteristics, evaluations of pain intensity were used as standard descriptive statistics (mean, standard deviation, and proportions). The qualitative variables were expressed as frequencies (N) and percentages (%). The relationships between qualitative variables were assessed with the chi-squared test. Significance was set at *p* < 0.05.

## 3. Results

### 3.1. Socio-Demographic Information

The examined group consisted of 100 females (48.5%) and 106 males (51.5%). Most of the 206 participants live in a big city that has more than 150,000 citizens. In an examined group, some people declared that they lived in a big city 37.9% (*n* = 78), some in a smaller city 29.6% (*n* = 61). Other respondents (32.5%) declared that they lived in villages (*n* = 67). All respondents were adults (Table 1).

The most numerous group consisted of people who had achieved a secondary education—31.1% (*n* = 64); 24.3% had achieved a higher level of education (*n* = 50). People who had completed vocational education represented 20.9% (*n* = 43). The two last possibilities were primary education—this option was chosen by 14.1% (*n* = 29)—and medical education, which was selected by 9.7% (*n* = 20).

### 3.2. Rating Pain

Patients were asked to rate pain in three stages. The first of these stages was to check the pain rating on a scale of pain during admission to the ED. The average value at the beginning was 6.31 (*n* = 180; SD = 2.09). When pain intensity was next checked when during the patient’s stay in ED, the average value was 4.4 (*n* = 180; SD = 2.37). The last check of the rate of pain was during discharge. The average rating was the lowest and was 2.89 (*n* = 179; SD = 2.44) (Table 2).

### 3.3. Dependence between the Rate of the Pain and Satisfaction with Provided Medical Service

In the end, the pain rating and satisfaction with the medical service were compared. At the time of discharge, 95% of patients who did not feel pain were satisfied with the provided medical service. There were slightly fewer people who experienced mild pain—92.1%. In the group of people declaring moderate pain, 80% of people were satisfied, and 63.9% of people with severe pain. There was no statistical dependence between the rate of pain and satisfaction with provided medical service (*p* = 0.056).

**Figure 1:**
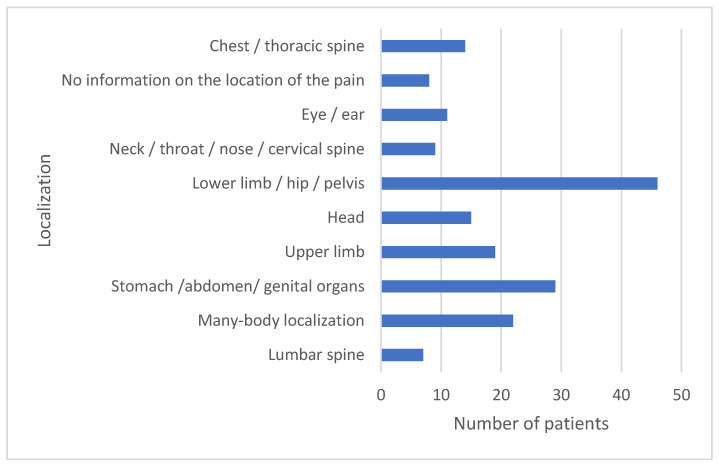
Localization of pain in people reporting to the hospital emergency department.

The three main reasons for reporting to the emergency department were pain localized at the following sites: lower limb/hip/pelvis (*n* = 46); stomach/abdomen/genital organs (*n* = 29); many-body localization (*n* = 22) (Figure 1).

## 4. Discussion

In this study, 86.89% (*n* = 179) of patients presented to the emergency department because of pain, which is a similar result as compared with other studies. [13,14,15]. Among the respondents, the mean level of pain intensity at hospital admission was 6.31 (SD = 2.09) Additionally, it decreased to a level of 2.89 (SD = 2.44) at discharge. Similar results in the significant reduction in pain intensity from the arrival to the hospital emergency department to discharge were obtained by Leigheb et al. [16].

When it came to patients’ satisfaction with receiving medical services, the majority of respondents were 86.41% (*n* = 178). Similar results were obtained by van Zanden et al. where patients included in the study expressed relatively high patient satisfaction with pain treatment—7.83 on a 0–10 scale [17].

Although there was a decrease in patients’ satisfaction with the services provided in the hospital emergency department with higher pain intensity (patients not feeling pain at discharge—94.12% satisfied; patients experiencing severe pain at discharge—63.91% satisfied), statistical significance was not demonstrated. Patients in emergency departments had very high expectations for pain relief, often much higher than people with postoperative pain [18].

Only 29.83% (*n* = 54) of patients had taken pain medication before arriving at the hospital emergency room. This result is similar to that of Tasdemir et al., where 30.1% of patients had taken pain medications before arriving on ED. However, in a study by Tasdemir et al., an additional 21.3% had used alternative pain treatments before arriving at the ED [19]. The causes of this condition can be complex and inconclusive. Thomason et al. showed that this may be due to patients’ beliefs that pain should be tolerated, as well as concerns about the side effects of antimicrobial drugs [20]. Moreover, another reason for not using painkillers before arriving at the emergency department may be the lack of knowledge about painkillers and their maximum daily doses, which was confirmed in their study by Fosnocht et al. and the limited knowledge about paracetamol [21].

### Study Limitations

The main limitations of this study were that it concerned only one hospital emergency department as well as the period of data collection, i.e., one month a year. Moreover, the small sample may limit the generalizability of the findings. Additionally, research was carried out during the COVID-19 pandemic, which considerably influenced disease and social-demographic information. Key limitations were the criteria of inclusion to the study and selected method of examination—survey/questionnaire in the paper form.

## 5. Conclusions

No statistical dependency between the results of analgesic treatments and the patient’s opinion has been observed. The majority of patients with pain discomfort visit emergency departments without looking for consultation in other locations or without taking analgesics. In the considered institution, patients were satisfied with the analgesic treatment, staff performance, and with the organization of the department. A low percentage of patients had taken pain medication before arriving at the hospital’s emergency department, a phenomenon of which the causes require further investigation. In addition, patient education in this regard could contribute to reducing the occupancy rate in hospital emergency departments.

## Figures and Tables

**Table 1:** Characteristics of the study population.

Variables	Sample *n* = 206
Sex (*n*; %)	
Male	106 (51.5%)
Female	100 (48.5%)
Age group (*n*; %)	
<30	76 (36.9%)
31–50	73 (35.4%)
51–70	30 (14.6%)
>71	27 (13.1%)
Place of residence (*n*; %)	
City larger than 150,000 citizens	78 (37.9%)
Smaller city	61 (29.6%)
Village	67 (32.5%)
Level of education	
Higher education	50 (24.3%)
Secondary education	64 (31.1%)
Vocational education	43 (20.9%)
Primary education	29 (14.1%)
Medical education	20 (9.7%)

**Table 2:** Pain rating.

Rating	Value
First check	6.31 (*n* = 180; SD = 2.09)
Second check	4.4 (*n* = 180; SD = 2.37)
Final check	2.89 (*n* = 179; SD = 2.44)

## Data Availability

The data presented in this study are available on request from the corresponding author.

## References

[1] Vedovetto A., Soriani N., Merlo E., Gregori D. (2014). The burden of inappropriate emergency department pediatric visits: Why Italy needs an urgent reform. Health Serv. Res..

[2] Uscher-Pines L., Pines J., Kellermann A., Gillen E., Mehrotra A. (2013). Emergency department visits for nonurgent conditions: Systematic literature review. Am. J. Manag. Care.

[3] Durgun H., Kaya H. (2018). The attitudes of emergency department nurses towards patient safety. Int. Emerg. Nurs..

[4] Cordell W.H., Keene K.K., Giles B.K., Jones J.B., Jones J.H., Brizendine E.J. (2002). The high prevalence of pain in emergency medical care. Am. J. Emerg. Med..

[5] Janati M., Kariman H., Memary E., Davarinezhad-Moghadam E., Arhami-Dolatabadi A. (2018). Educational Intervention Effect on Pain Management Quality in Emergency Department; a Clinical Audit. Adv. J. Emerg. Med..

[6] e Silva L.O.J., Scherber K., Cabrera D., Motov S., Erwin P.J., West C.P., Murad M.H., Bellolio M.F. (2018). Safety and Efficacy of Intravenous Lidocaine for Pain Management in the Emergency Department: A Systematic Review. Ann. Emerg. Med..

[7] Kacprzyk A., Stefura T., Chlopas K., Trzeciak K., Zalustowicz A., Rubinkiewicz M., Pedziwiatr M., Rembiasz K., Major P. (2020). “Analysis of readmissions to the emergency department among patients presenting with abdominal pain”. BMC Emerg. Med..

[8] Soremekun O.A., Takayesu J.K., Bohan S.J. (2011). Framework for analyzing wait times and other factors that impact patient satisfaction in the emergency department. J. Emerg. Med..

[9] Abdolrazaghnejad A., Banaie M., Tavakoli N., Safdari M., Rajabpour-Sanati A. (2018). Pain Management in the Emergency Department: A Review Article on Options and Methods. Adv. J. Emerg. Med..

[10] Aiello A., Garman A., Morris S.B. (2003). Patient satisfaction with nursing care: A multilevel analysis. Qual. Manag. Health Care.

[11] Haefeli M., Elfering A. (2006). Pain assessment. Eur. Spine J..

[12] Anekar A.A., Cascella M. (2021). WHO Analgesic Ladder. StatPearls.

[13] Berben S.A., Meijs T.H., van Dongen R.T., van Vugt A.B., Vloet L.C., Mintjes-de Groot J.J., van Achterberg T. (2008). Pain prevalence and pain relief in trauma patients in the Accident & Emergency department. Injury.

[14] Gueant S., Taleb A., Borel-Kuhner J., Cauterman M., Raphael M., Nathan G., Ricard-Hibon A. (2011). Quality of pain management in the emergency department: Results of a multicentre prospective study. Eur. J. Anaesthesiol..

[15] Chang H.Y., Daubresse M., Kruszewski S.P., Alexander G.C. (2014). Prevalence and treatment of pain in EDs in the United States, 2000 to 2010. Am. J. Emerg. Med..

[16] Leigheb M., Sabbatini M., Baldrighi M., Hasenboehler E.A., Briacca L., Grassi F., Cannas M., Avanzi G., Castello L.M. (2017). Prospective analysis of pain and pain management in an emergency department. Acta Biomed..

[17] van Zanden J.E., Wagenaar S., Ter Maaten J.M., Ter Maaten J.C., Ligtenberg J.J.M. (2018). Pain score, desire for pain treatment and effect on pain satisfaction in the emergency department: A prospective, observational study. BMC Emerg. Med..

[18] Motov S.M., Khan A.N. (2008). Problems and barriers of pain management in the emergency department: Are we ever going to get better?. J. Pain Res..

[19] Tasdemir N., Celik S. (2016). Self-reported pain relief interventions of patients before emergency department arrival. Int. Emerg. Nurs..

[20] Thomason T.E., McCune J.S., Bernard S.A., Winer E.P., Tremont S., Lindley C.M. (1998). Cancer pain survey: Patient-centered issues in control. J. Pain Symptom Manag..

[21] Fosnocht D., Taylor J.R., Caravati E.M. (2008). Emergency department patient knowledge concerning acetaminophen (paracetamol) in over-the-counter and prescription analgesics. Emerg. Med. J..

